# Intractable itch relieved by 4-phenylbutyrate therapy in patients with progressive familial intrahepatic cholestasis type 1

**DOI:** 10.1186/1750-1172-9-89

**Published:** 2014-07-15

**Authors:** Yasuhiro Hasegawa, Hisamitsu Hayashi, Sotaro Naoi, Hiroki Kondou, Kazuhiko Bessho, Koji Igarashi, Kentaro Hanada, Kie Nakao, Takeshi Kimura, Akiko Konishi, Hironori Nagasaka, Yoko Miyoshi, Keiichi Ozono, Hiroyuki Kusuhara

**Affiliations:** 1 Department of Pediatrics, Osaka University Graduate School of Medicine, 2-2 Yamada-oka, Suita, Osaka 565-0871, Japan; 2 Laboratory of Molecular Pharmacokinetics, Graduate School of Pharmaceutical Sciences, The University of Tokyo, 7-3-1 Hongo, Bunkyo-ku, Tokyo 113-0033, Japan; 3 Bioscience Division, Reagent Development Department, TOSOH Corporation, 2743-1 Hayakawa, Ayase-shi, Kanagawa 252-1123, Japan; 4 Department of Biochemistry and Cell Biology, National Institute of Infectious Diseases, 1-23-1, Toyama, Shinjuku-ku, Tokyo 162-8640, Japan; 5 Department of Pediatrics, Takarazuka City Hospital, 4-5-1 Kohama, Takarazuka-shi, Hyogo 665-0827, Japan

**Keywords:** Pediatric liver disease, Cholestasis, PFIC1, Pruritus, 4PB

## Abstract

**Background:**

Progressive familial intrahepatic cholestasis type 1 (PFIC1), an inherited liver disease caused by mutations in *ATP8B1*, progresses to severe cholestasis with a sustained intractable itch. Currently, no effective therapy has been established for PFIC1. Decreased function of the bile salt export pump (BSEP) in hepatocytes is suggested to be responsible for the severe cholestasis observed in PFIC1. We found a previously unidentified pharmacological effect of 4-phenylbutyrate (4PB) that increases the expression and function of BSEP. Here, we tested 4PB therapy in three patients with PFIC1.

**Methods:**

The therapeutic potency of 4PB in these patients was tested by oral administration of this drug with gradually increasing dosage (200, 350, and 500 mg/kg/day) for 6 months. Biochemical, histological, and clinical data were collected.

**Results:**

4PB therapy had no beneficial effect on the patients’ liver functions, as assessed by biochemical and histological analyses, despite an increase in hepatic BSEP expression. However, therapy with 4PB at a dosage of 350 or 500 mg/kg/day significantly relieved the intractable itch. Serum levels of potential pruritogens in cholestasis were much higher than the reference ranges during the 4PB therapy.

**Conclusions:**

4PB therapy may be a new medication for patients with intractable cholestatic pruritus and may improve quality of life for patients and their families.

## Background

Progressive familial intrahepatic cholestasis type 1 (PFIC1), a rare inherited autosomal recessive liver disease caused by mutations in *ATP8B1*, is characterized primarily by normal serum gamma-glutamyl transferase (GGT), intrahepatic cholestasis and jaundice in the first year of life [1]. This disease progresses to severe cholestasis with sustained intractable itching, jaundice, watery diarrhea, failure to thrive, pancreatitis, and deafness, resulting in liver failure and death before adulthood [2]. The main complaint in the clinical course of patients with PFIC1 is often intractable itching, which significantly disrupts the patients’ ability to sleep and thus decreases the quality of life of patients and their families [3]. No effective medical therapy for this disease is currently available [3]. Even liver transplantation is insufficient to improve the clinical course and outcomes of patients with PFIC1 because of steatosis and fibrosis [4].

ATP8B1 is a member of the P4 subfamily of P-type adenosine triphosphatases and is expressed on the apical membrane of many epithelial cells including hepatocytes. ATP8B1 translocates phosphatidylserine (PS) from the outer leaflet to the inner leaflet and thereby contributes to making the hepatocanalicular membrane (CM) a rigid, liquid-ordered membrane [5,6]. In patients with PFIC1, the well-organized aminophospholipid asymmetry of the CM is disrupted by the impaired function of ATP8B1, leading to a decrease in the transport activity of the bile salt export pump (BSEP), an ABC transporter that is localized on the CM and that predominantly mediates biliary excretion of bile salts [7-10], and subsequently to the onset of severe intrahepatic cholestasis [11]. Alternatively, in patients with PFIC1, nuclear translocation of the farnesoid X receptor (FXR), a transcription factor that controls bile acid homeostasis, is disrupted and causes a decrease in BSEP expression at the CM because of mass action related to the decreased expression of BSEP mRNA [12]. Therefore, in either cause of PFIC1, an increase in BSEP function is expected to compensate for the reduced capacity for bile salt excretion into bile in patients with PFIC1, and may improve their liver function.

We have published experimental evidence that 4-phenylbutyrate (4PB), a drug used to treat ornithine transcarbamylase deficiency (OTCD), has another newly identified pharmacological effect that increases the hepatocanalicular expression of BSEP and the hepatocyte capacity for biliary excretion of bile salts when given at a clinically relevant concentration in OTCD patients [13]. The higher BSEP expression level in liver specimens from patients with OTCD after 4PB therapy compared with that before 4PB therapy suggests that 4PB treatment increases BSEP expression in humans [14]. Furthermore, our group and Gonzales *et al.* reported recently that 4PB therapy restored decreased BSEP expression, improved liver functions in histological and biochemical analysis, and relieved intractable pruritus in patients with PFIC type 2 (PFIC2), an inherited autosomal recessive liver disease caused by mutations in *BSEP*[15,16]. PFIC2 patients present with similar clinical symptoms and biological parameters as PFIC1 patients [17,18]. Together, these results suggest the possibility that 4PB may be a potential therapeutic compound for PFIC1 patients that could act to restore the reduced capacity of biliary excretion of bile salt through increasing BSEP expression on the CM.

To test this hypothesis, our current study investigated the effects of 4PB therapy in three PFIC1 patients. 4PB was administered orally with gradually increasing dosage (200, 350, and 500 mg/kg/day) for 6 months. We collected data on serum liver tests, histological analyses, pruritus score, and the clinical course for these patients.

## Methods

We obtained approval for the study from the institutional ethics review boards. Informed consent was obtained from the patients’ parents before assessment because the patients were younger than 18 years of age. A detailed description of the materials and methods is presented in the Additional file 1. All materials and methods used standard techniques and commercially available reagents.

### Patients

The patients enrolled in our study were three Japanese boys (Patient 1, 2, and 3) who were seen at Osaka University Hospital in 2012 and aged 2-, 6-, and 16-years old, respectively. All three patients developed hepatocellular cholestasis with mild elevation of serum aspartate aminotransaminase (AST) and alanine amino transaminase (ALT) levels and normal GGT levels as infants and experienced sleep disturbance because of intractable itch at around 4, 6, and 4 months of age, respectively. Patient 3 had difficulty in getting to sleep by the end of elementary school. Despite treatment with drugs including ursodeoxycholic acid, topical steroids, and antihistamine agents for 1.5, 5, and 15 years in patient 1, 2, and 3, respectively, these patients continued to experience severe cholestasis with sustained intractable itch, jaundice, diarrhea, and failure to thrive, which are typical clinical symptoms of PFIC1. The patients and/or their families preferred medical treatment to surgical procedure like partial external biliary diversion. Therefore, the patients were enrolled in this clinical study. The administration of original drugs was maintained during and after the course of 4PB treatment. The drugs given to the patients before, during, and after the course of this study are listed in the Additional file 1: Table S1.

### Sequence analysis of *ATP8B1* and *ABCB11*

Genomic DNA was isolated from peripheral blood leukocytes using a Wizard Genomic DNA Purification Kit (Promega, Madison, WI), and all exons of *ATP8B1* and *ABCB11* and flanking intron–exon boundaries were analyzed as described previously [16,19,20].

### Treatment of PFIC1 patients with 4PB

Oral administration of 4PB (Ammonaps; Swedish Orphan Inter AB., Stockholm, Sweden) was started at a daily dosage of 200 mg/kg/day divided into four doses a day. After 1 month, the dosage was increased to 350 mg/kg/day and this was maintained for an additional month. Because neither a therapeutic effect nor any side effects were observed, the dosage was increased to 500 mg/kg/day, which is the clinically relevant dosage for OTCD, and this dosage was maintained for the next 4 months. A liver biopsy sample was collected 1 day before and after the course of 4PB treatment. A part of the sample was preserved in RNAlater (Qiagen, Hilden, Germany) for RNA preparation and stored at -20°C. Another portion was fixed in 10% formaldehyde at room temperature for histological analysis, and the remaining portion was snap-frozen in liquid nitrogen for preparation of membrane fractions and stored at -70°C in a deep freezer. Serum was collected before, during, and after the course of 4PB treatment. Liver function tests were performed using standard methods immediately after collection, and the remaining specimens were preserved at -70°C for further analysis.

### Pruritus evaluation

Pruritus severity was scored as reported previously [21]: 0, none; 1, mild scratching when undistracted; 2, active scratching without abrasion; 3, abrasions; or 4, cutaneous mutilation, with bleeding and scarring.

### Quantitative determination of pruritogen levels in serum

The concentration and activity of autotaxin (ATX) in serum were assessed using a specific two-site enzyme immunoassay and the measurement of choline liberated from the substrate lysophosphatidylcholine as described previously [16,22].

### Histological analysis of the patients’ liver specimens

Liver biopsy specimens were fixed in 10% formalin and embedded in paraffin. Four-micrometer-thick sections were prepared from the liver specimens and subjected to hematoxylin-eosin (HE) staining and immunohistochemistry followed by microscopic analysis with an Olympus CX41 or Olympus BX40 microscope (Olympus, Tokyo, Japan) to evaluate the degree of cholestasis, fibrosis, and inflammation in the liver tissues.

### Preparation of crude membrane, nuclear, and cytosolic fractions from the patients’ liver specimens

Liver specimens from the patients were homogenized in hypotonic buffer (1 mM EDTA, 5 mM sodium phosphate, pH 7.0) supplemented with protease inhibitor cocktails (Sigma-Aldrich, St. Louis, MO) using a QIAshredder (Qiagen), and then centrifuged at 800 × g for 10 min at 4°C. The supernatant was ultracentrifuged at 100,000 × g for 1 h at 4°C and the pellet and supernatant were used as the crude membrane and cytosolic fractions, respectively. After centrifugation at 800 × g, the pellet was suspended with high salt buffer (20 mM Tris–HCl pH 7.9, 400 mM NaCl, 0.1 mM EDTA, 0.1 mM EGTA Na, 0.1% NP-40, 1 mM DTT, 10% glycerol, 0.1% protease inhibitor cocktail), incubated on ice for 50 min with vortex mixing every 10 min, and centrifuged at 3000 × g for 10 min at 4°C, and the supernatant was used as the nuclear extract.

### Immunoblotting

Specimens were loaded into each well of a 7% SDS-PAGE plate with a 3.75% stacking gel, and subjected to immunoblotting as described previously [13,14,23]. Immunoreactivity was detected with an ECL Advance™ Western Blotting Detection Kit (Amersham Biosciences, Piscataway, NJ). The intensity of the band was quantified by MultiGauge software (version 2.0; Fujifilm, Tokyo, Japan). Expression levels of ATP8B1, BSEP, and CDC50A were normalized by the expression of Na^+^, K^+^-ATPase α1 subunit (NaKα1), which was not affected by the treatment with 4PB (data not shown).

## Results

### Diagnosis of PFIC1 in the patients

**Figure 1:**
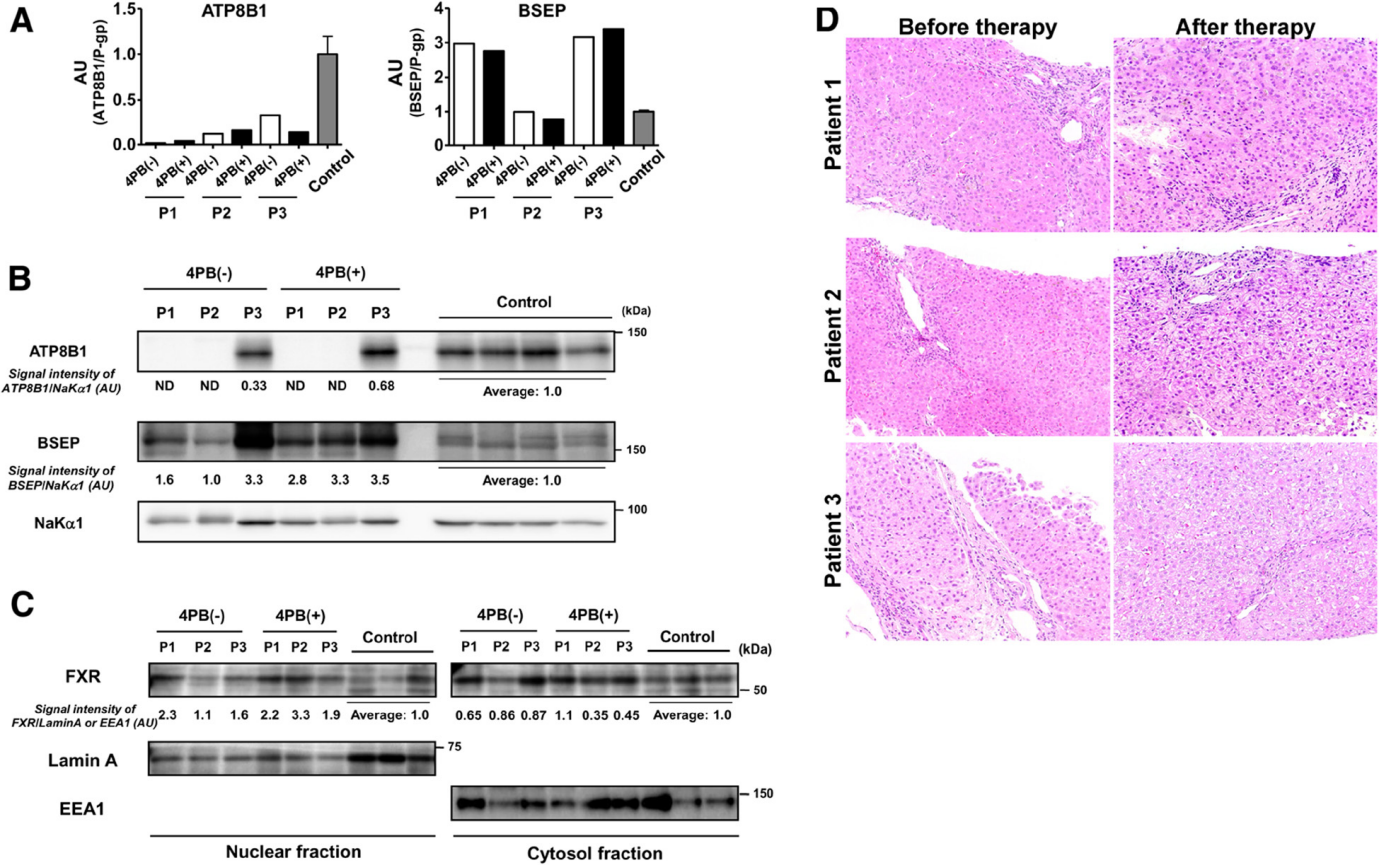
**Studies of liver biopsy specimens obtained from the patients before and after 4PB therapy. (D)** Histological characteristics of liver sections. Liver sections prepared from the liver biopsy specimens of the patients before and after the 4PB therapy were subjected to HE staining as described in Patients and Methods. A typical image under each condition is shown. Original magnification; 200×. **(A-C)** Analysis of mRNA and protein expression levels. cDNA **(A)**, crude membrane fractions **(B)**, and nuclear and cytosolic fractions **(C)** were prepared from liver biopsy specimens from the patients and then analyzed by qPCR **(A)** and immunoblotting **(B and C)** as described in Materials and Methods. In **(A)**, the data were obtained from triplicate determination. Each bar represents the mean ± SE of individual specimens. In **(B and C)**, the signal intensity of ATP8B1, BSEP, and FXR relative to that of NaKα1, LaminA, and EEA1 is presented below each panel. AU, arbitrary unit; P-gp, P-glycoprotein; P1, patient 1; P2, patient 2; P3, patient 3; 4PB(-), before 4PB treatment; 4PB(+), after 4PB treatment.

Sequencing analysis of all encoding exons and flanking intron–exon boundaries of *ATP8B1* identified a heterozygous mutation c.3033–34del (frame shift or splicing defect) in patient 1 and a heterozygous mutation c.1587–89del (p.F529del) in patient 2 (Table 1). The c.1587–89del (p.F529del) mutation has been reported previously in European PFIC1 patients of Caucasian descent [19]. Although no other mutations were found in *ATP8B1* on the other allele, as was the case for several PFIC1 patients reported in a previous study [19], both patients were diagnosed with PFIC1 because they exhibited the typical clinical symptoms of PFIC1 and because of the low mRNA expression and no detectable protein expression of ATP8B1 in their liver biopsy specimens (Figure 1A, B). The near absence and marked decrease of hepatic ATP8B1 mRNA in patients 1 and 2 may be explained by other mutations in the promoter region and/or untranslated region (UTR) of *ATP8B1* on the other allele that affect transcription of *ATP8B1* and/or stabilization of ATP8B1 mRNA. Patient 3 was diagnosed with PFIC1 because of lower mRNA and protein expression of ATP8B1 in his liver specimen (Figure 1A, B) and because of his clinical symptoms including intrahepatic cholestasis with normal GGT, intractable itching, failure to thrive, and deafness. The sequencing analysis of *ATP8B1* in patient 3 identified c.234C > G (p.H78Q) (rs3745079) and c.2021 T > C (p.M674T) (rs35470719) mutations on one allele and a mutation c.1729A > G (p.I577V) (rs3745078) on the other allele. However, given that these mutations had no significant effect on mRNA and protein expression, trafficking to the plasma membrane, and PS flippase activity of ATP8B1 in *in vitro* studies (Additional file 2: Figure S1 A–D), it is likely that the decreased mRNA and protein expression of ATP8B1 in this patient is caused by mutations in the promoter region and/or UTR of *ATP8B1* that affect transcription of *ATP8B1* and stabilization of ATP8B1 mRNA, but not by the mutations analyzed in this study.

**Table 1:** ATP8B1 mutation and biochemical parameters in patients enrolled in this study

	**Sex**	**Age**	***ATP8B1 *** **mutation in allele 1**	***ATP8B1 *** **mutation in allele 2**	**AST/ALT/GGT (U/L)**	**T-Bil/D-Bil/BA (μM)**	**Pruritus score**
Patient 1	M	2	c. 3033-3034del (Frame shift)	Not found	83/60/36	4.8/4.6/301.3	4
Patient 2	M	6	c.1585-1587TTCdel (p.F529del)	Not found	81/15/27	3.4/3.2/214.1	4
Patient 3	M	16	c.234C > G (p.H78Q) c.202IT > C (p.M674T)	c.1729A > G (p.I577V)	63/51/19	0.6/0.3/54.6	4

### Therapeutic effect of 4PB in PFIC1 patients

**Figure 2:**
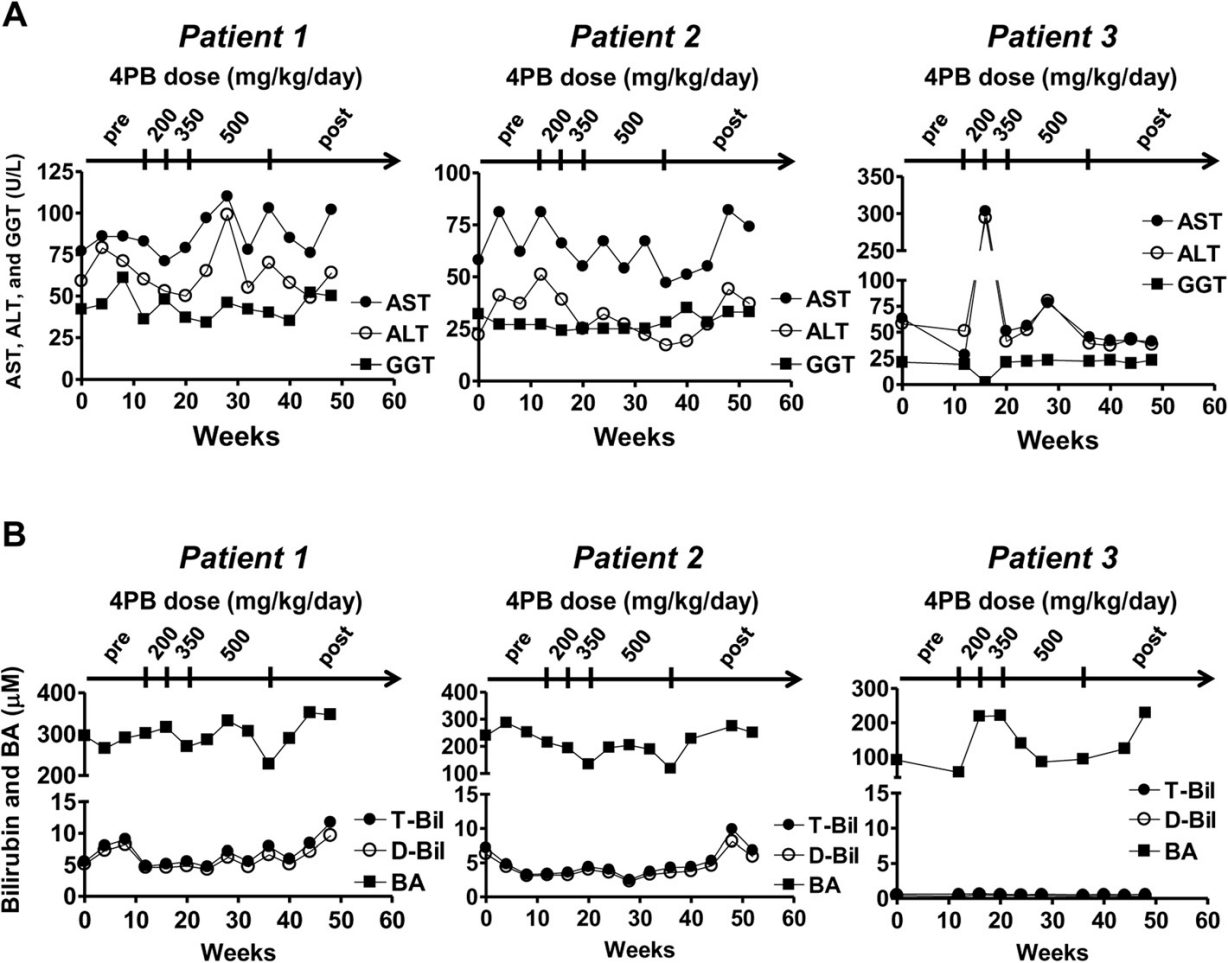
**Liver function testing before, during and after the course of 4PB therapy in the patients.** Serum AST, ALT, GGT **(A)**, total bilirubin, direct bilirubin, and bile acids **(B)** levels were monitored before, during and after the 4PB therapy. T-Bil, total bilirubin; D-Bil, direct bilirubin; BA, bile acids.

**Figure 3:**
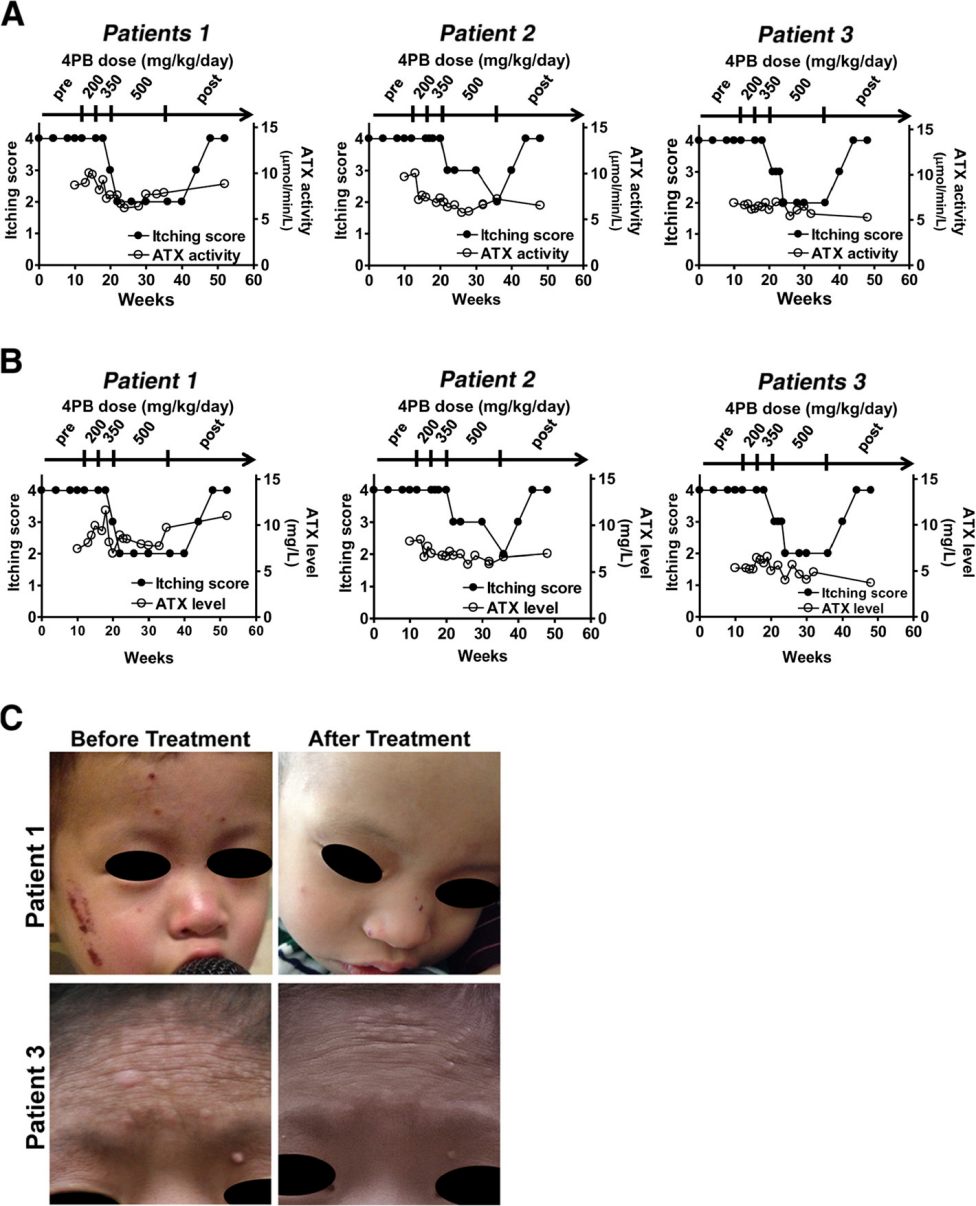
**Itching intensity in the patients before, during, and after the course of 4PB therapy. (A, B)** Correlation diagram of itching scores for the PFIC1 patients with serum ATX activity **(A)** and ATX level **(B)** in the patients before, during, and after 4PB therapy. Pruritus severity was scored ranging from 0 (no pruritus) to 4 (cutaneous mutilation, with bleeding and scarring) as described in Patients and Methods. **(C)** Skin of the patients before and after the 4PB therapy.

During the period of 4PB treatment, no improvement was observed in liver function tests for any of the patients (Figure 2). However, their itching started to attenuate in patient 1 three weeks after the dosage of 4PB was increased to 350 mg/kg/day, in patient 2 one week after the dosage was increased to 500 mg/kg/day, and in patient 3 four weeks after the dosage of 4PB was increased to 350 mg/kg/day. The itching score declined from 4 to 2 in all patients (Figure 3A, B). Although there were multiple dark erosions in the skin of all patients and elephantiasis in patient 3 due to intense and continual scratching, after the onset of 4PB treatment at the dosage of 350 or 500 mg/kg/day, the frequency and intensity of skin scratching was markedly decreased, leading to diminished skin erosion and hemorrhage and improved skin appearance (Figure 3C). At the end of the therapy, hemorrhage and eschar on the patients’ skin were diminished and areas of fresh normal skin were evident. The parents of the patients noted an improvement in sleep disturbance during the night and in their child’s skin condition. In contrast to the relief of the itching, the serum levels of bile acids and ATX and of ATX activity, all of which have been proposed as potential pruritogens in cholestasis [24], were not decreased by 4PB therapy in any of the patients (Figures 2B, 3A, B). The itch remained unchanged for 6, 4, and 6 weeks after cessation of 4PB therapy in patients 1, 2, and 3, respectively, but then gradually exacerbated, resulting in regeneration of erosion and hemorrhage again because of intense scratching. In all the patients, 8 weeks after the end of 4PB therapy, the itching score returned to values equal to those before the treatment (Figure 3A, B). Because of the bad taste of 4PB, all patients had difficulty taking the doses at the beginning of the therapy. However, their parents noted the improvement in their child’s sleep during the night and in the skin conditions, and encouraged their child to continue the therapy. No patients dropped out of this study. No severe side effects were observed during and after the 4PB therapy. The temporary elevation of AST and ALT concentrations after patient 3 began 4PB treatment at 200 mg/kg/day was thought to be caused by adenovirus infection and not by any adverse effect of the 4PB therapy, because both markers increased promptly after the adenovirus infection appeared and decreased to the basal levels concurrently with recovery from the infection (Figure 2A).

### Effect of 4PB therapy on liver histology and BSEP expression in PFIC1 patients

A liver biopsy was performed 6 months after the initiation of 4PB therapy and compared with the specimens obtained 1 day before onset of 4PB therapy. The specimens of the age-matched control subjects were obtained from OTCD patients without administration of 4PB when they underwent liver transplantation. qPCR and immunoblot analysis demonstrated that even in the specimens taken after the 4PB therapy, the mRNA expression of ATP8B1 was still much lower in all the patients than that of age-matched control subjects, and ATP8B1 protein expression in the membrane fraction was undetectable in patients 1 and 2. In patient 3, ATP8B1 expression was increased about 2-fold by 4PB therapy, but still lower than that in age-matched control subjects (Figure 1B, C). This result was consistent with the lack of change in ATP8B1–FLAG ^F529del^ and a 2.1- and 1.3-fold increase in ATP8B1^H78Q+M674T^–FLAG and ATP8B1^I577V^–FLAG in UPS-1 cells after treatment with 4PB at a clinically relevant concentration (1 mM) (Additional file 3: Figure S2). BSEP protein expression was increased after 4PB therapy without affecting its mRNA expression as is the case in patients with PFIC2 and OTCD (Figure 1A, B) [14,16]. The amount of FXR in the nuclear fraction of cells from the PFIC1 patients was nearly equal to or a little higher than that of age-matched control subjects, and was not significantly affected by 4PB therapy except for patient 2 (Figure 1C). In this patient, the amount of FXR was increased 3-fold after 4PB therapy. Histological analysis showed that in the specimens obtained from the PFIC1 patients before this study, the portal area was enlarged and had progressed to fibrosis with mild inflammation and partly bridging fibrosis and that 4PB therapy caused no remarkable change in these features (Figure 1D).

## Discussion

The main complaint in the clinical course of PFIC1 is often the intractable itching, which significantly disrupts the patients’ activities of daily living, work productivity, and ability to sleep, and thereby decreases the quality of life for them and their families [2]. Topical steroids, antihistamine agents, and rifampicin are the only prescribed drugs currently available for the cholestatic pruritus in PFIC1 patients, but these medications are often ineffective, as was the case for the patients enrolled in this study [3]. The principal finding of our current study is that 4PB therapy at a clinically relevant dosage used in OTCD patients markedly relieved intractable cholestatic pruritus in PFIC1 patients. At the end of the therapy, hemorrhage and eschar on the patients’ skin were diminished, and areas of fresh normal skin appeared as the frequency and intensity of skin scratching decreased. Parents of the patients noted that 4PB therapy made it easier for their child to get to sleep and markedly reduced their child’s sleep disturbance during the night. Thus, favorable outcomes of 4PB therapy were observed, and these were dose-dependent, although the patients and their families in this clinical study were not informed of the detailed protocol. The facts that the patients and their families were not informed of this aspect and that the drugs prescribed for 1.5, 5, and 15 years in patient 1, 2, and 3, respectively, before this study were ineffective in improving the sustained intractable itch suggest that the relief of refractory cholestatic itching in these PFIC1 patients occurred because of the 4PB therapy and not because of a placebo effect or an effect of other medications.

The visual analog scale (VAS) is the most general method for assessing pruritus, but is not easily applied to younger children because VAS is a graphic method based on the patients’ subjective rating of symptoms [25]. Therefore, in this study, the patients’ itching was evaluated on the basis of cutaneous findings and scored according to the method used in a previous report [21]. A potential concern when using this method is that the improvement in itch may be underestimated. The severity of skin erosion, thickness, and cicatrices depends on the duration of the itch and can vary in the patients scored as 4. Therefore, there may have been a time lag in the improvements in skin appearance even if the frequency and intensity of scratching decreased to the same degree in all patients. In this study, the improvement of pruritus in patient 3, who experienced intractable pruritus for a longer period than patients 1 and 2, might have been underestimated compared with that in patients 1 and 2. To overcome this limitation, it might be better to apply the 5-D itch scale, a recently developed method that assesses the subjective symptoms of itching in patients from five dimensions: degree, duration, direction, disability and distribution [25]. In the 5-D itch scale, but not in the VAS, the subjective symptoms reported by the patient can be supported by his/her family. Therefore, in future clinical studies, the change in chronic pruritus in younger patients should be explored using the 5-D itch scale as well as the method used in this study. This should allow a more accurate determination of the clinical outcomes and the beneficial effects of 4PB therapy on cholestatic pruritus in PFIC1 patients.

In contrast to the relief of intractable cholestatic itching, beneficial effects of 4PB therapy were not observed in liver function tests and liver histology despite an increase in BSEP expression in liver membrane fractions as reported previously (Figure 1B, D and 2) [14,16]. This could be because a two- to three-fold increase in BSEP expression may be insufficient to improve intrahepatic cholestasis in PFIC1 patients. The other possible reason is that the transport activity of BSEP is lost completely in PFIC1 patients because of the disrupted lipid asymmetry of the CM [6,11] and, consequently, the increase in BSEP expression by 4PB therapy cannot compensate for the reduced capacity of bile salt excretion into bile. Although no remarkable improvement in liver function was observed in the patients in our study, 4PB therapy might have therapeutic potency for specific PFIC1 patients with mutations in *ATP8B1* that attenuate ATP8B1 expression, but do not affect its protein activity. An *in vitro* analysis has shown that treatment with 4PB partially restored the decreased expression of ATP8B1 caused by p.G308V, p.D454G, and p.D544N, all of which are naturally occurring mutations [26]. Future clinical studies should validate the therapeutic effect of 4PB and its safety for use in PFIC1 patients who carry mutations that attenuate ATP8B1 expression but do not affect its protein activity.

At present, the mechanism underlying the relief of cholestatic pruritus by 4PB therapy remains to be elucidated. No decrease in the factors suspected to be causally associated with cholestatic pruritus (Figures 2, 3A, B) are consistent with the observation in PFIC2 patient during 4PB therapy [16]. 4PB and/or its metabolites may modulate the local concentrations of these pruritogens, which may not have been detected by systemic measurements. The physiological function of ATX, an enzyme secreted extracellularly that generates lysophosphatidic acid, is thought to be mediated predominantly by activation of G protein-coupled receptors (GPCRs) [27]. TGR5, a GPCR activated by bile salts, in sensory nerves could contribute to bile salt-induced itching [28]; if so, 4PB and/or its metabolites might antagonize the GPCRs responsible for itch signaling and therefore attenuate the activation of sensory neurons. Alternatively, 4PB therapy may disrupt pruriceptive projections to the brain through distribution of 4PB and/or its metabolites into the brain [29] or may affect pruritogens or anti-pruritogens that have not been identified yet. Further studies to test these possibilities will provide a better understanding of the mechanisms responsible for the effects of 4PB therapy on cholestatic pruritus and thereby the molecular mechanism of cholestatic pruritus itself. Information obtained from these studies will contribute to the development of new molecular target drugs for cholestatic pruritus, which will hopefully be more effective than 4PB and consequently, improve the clinical application of 4PB.

## Conclusions

Our study has provided clinical evidence that 4PB therapy can relieve the refractory itching in PFIC1 patients, and thereby improved the quality of life of the patients and their families. Future clinical studies with more patients and longer time periods than were possible in this study should be undertaken to confirm the favorable effects of 4PB therapy using the 5-D itch scale and the other method used in this study. If confirmed, 4PB therapy could become the preferred choice, instead of topical steroids, antihistamine agents, and surgical procedures, for attenuating cholestatic pruritus in patients with PFIC1 and benign intrahepatic cholestasis type 1 (BRIC1), a hereditary disorder characterized by mutations in *ATP8B1* and by recurrent and intermittent episodes of cholestasis with refractory cholestatic pruritus [30]. 4PB therapy might also be effective for intractable pruritus caused by other cholestatic disorders such as Alagille syndrome. Clinical trials will be required to determine the utility and safety of 4PB as a therapy for these diseases.

## Abbreviations

ALT: Alanine amino transaminase; AST: Aspartate aminotransaminase; ATX: Autotaxin; AU: Arbitrary units; BA: Bile acids; BRIC: Benign intrahepatic cholestasis; BSEP: Bile salt export pump; CM: Canalicular membrane; D-Bil: Direct bilirubin; ER: Endoplasmic reticulum; EV: Empty vector; FITC: Fluorescein isothiocyanate; FXR: Farnesoid X receptor; GGT: Gamma-glutamyl transferase; GPCR: G protein-coupled receptor; HA: Hemagglutinin antigen; HE: Hematoxylin-eosin; Na^+^: K^+^-ATPase α1 subunit, NaKα1; ND: Not detected because of low expression; OTCD: Ornithine transcarbamylase deficiency; PFIC: Progressive familial intrahepatic cholestasis; PS: Phosphatidylserine; qPCR: Quantitative polymerase chain reaction; SE: Standard error; T-Bil: Total bilirubin; VAS: Visual analog scale; WT: Wild type; 4PB: 4-phenylbutyrate.

## Competing interests

H.H. has applied a patent on the effect of 4-phenylbutyrate on bile salt export pump (US serial No.13/299,989).

## Author contribution

YH recruited and enrolled the patients, collected specimens from the patients, performed the clinical assessment and follow-up, contributed to data interpretation, and drafted the manuscript together with HH. HH directed and supervised all of the research and took a lead role in writing the manuscript. SN performed most of the *in vitro* experiments, contributed to data interpretation, and drafted the manuscript together with HH. HK and KB recruited and enrolled the patients, collected specimens from the patients, carried out clinical assessment and follow-up, contributed to data interpretation, and revised the manuscript for intellectual content. KI measured ATX concentration. KH provided UPS-1 cells. KN, TK, and AK collected specimens from the patients and carried out clinical assessment and follow-up. HN helped to make a diagnosis of the patients, and contributed to data interpretation. YM, KO, and HK contributed to data interpretation and revised the manuscript for intellectual content. All authors approved the manuscript before submission.

## Supplementary Material

Additional file 1 Supplemental information. Click here for file

Additional file 2 **Effects of mutations in *ATP8B1 *** **on mRNA and protein expression levels, cellular localization, and function of ATP8B1.** Click here for file

Additional file 3 Effect of 4PB on the expression levels of ATP8B1 mutants. Click here for file

## References

[B1] Bull LN van Eijk MJ Pawlikowska L DeYoung JA Juijn JA Liao M Klomp LW Lomri N Berger R Scharschmidt BF Knisely AS Houwen RH Freimer NB A gene encoding a P-type ATPase mutated in two forms of hereditary cholestasis Nat Genet 1998 18 3 219 224 10.1038/ng0398-219 9500542

[B2] Morotti RA Suchy FJ Magid MS Progressive familial intrahepatic cholestasis (PFIC) type 1, 2, and 3: a review of the liver pathology findings Semin Liver Dis 2011 31 1 3 10 10.1055/s-0031-1272831 21344347

[B3] Suchy FJ Shneider BL 14. familial hepatocellular cholestasis Liver Disease in Children 3rd Edition 2007 310 314

[B4] Hori T Egawa H Takada Y Ueda M Oike F Ogura Y Sakamoto S Kasahara M Ogawa K Miyagawa-Hayashino A Yonekawa Y Yorifuji T Watanabe K Doi H Nguyen JH Chen F Baine AM Gardner LB Uemoto S Progressive familial intrahepatic cholestasis: a single-center experience of living-donor liver transplantation during two decades in Japan Clin Transplant 2011 25 5 776 785 10.1111/j.1399-0012.2010.01368.x 21158920

[B5] Paulusma CC Folmer DE Ho-Mok KS de Waart DR Hilarius PM Verhoeven AJ Oude Elferink RP ATP8B1 requires an accessory protein for endoplasmic reticulum exit and plasma membrane lipid flippase activity Hepatology 2008 47 1 268 278 17948906 10.1002/hep.21950

[B6] Paulusma CC Groen A Kunne C Ho-Mok KS Spijkerboer AL Rudi de Waart D Hoek FJ Vreeling H Hoeben KA van Marle J Pawlikowska L Bull LN Hofmann AF Knisely AS Oude Elferink RP Atp8b1 deficiency in mice reduces resistance of the canalicular membrane to hydrophobic bile salts and impairs bile salt transport Hepatology 2006 44 1 195 204 10.1002/hep.21212 16799980

[B7] Byrne JA Strautnieks SS Mieli-Vergani G Higgins CF Linton KJ Thompson RJ The human bile salt export pump: characterization of substrate specificity and identification of inhibitors Gastroenterology 2002 123 5 1649 1658 10.1053/gast.2002.36591 12404239

[B8] Gerloff T Stieger B Hagenbuch B Madon J Landmann L Roth J Hofmann AF Meier PJ The sister of P-glycoprotein represents the canalicular bile salt export pump of mammalian liver J Biol Chem 1998 273 16 10046 10050 10.1074/jbc.273.16.10046 9545351

[B9] Hayashi H Takada T Suzuki H Onuki R Hofmann AF Sugiyama Y Transport by vesicles of glycine- and taurine-conjugated bile salts and taurolithocholate 3-sulfate: a comparison of human BSEP with rat Bsep Biochim Biophys Acta 2005 1738 1–3 54 62 16332456 10.1016/j.bbalip.2005.10.006

[B10] Noe J Stieger B Meier PJ Functional expression of the canalicular bile salt export pump of human liver Gastroenterology 2002 123 5 1659 1666 10.1053/gast.2002.36587 12404240

[B11] Paulusma CC de Waart DR Kunne C Mok KS Elferink RP Activity of the bile salt export pump (ABCB11) is critically dependent on canalicular membrane cholesterol content J Biol Chem 2009 284 15 9947 9954 10.1074/jbc.M808667200 19228692 PMC2665118

[B12] Chen F Ananthanarayanan M Emre S Neimark E Bull LN Knisely AS Strautnieks SS Thompson RJ Magid MS Gordon R Balasubramanian N Suchy FJ Shneider BL Progressive familial intrahepatic cholestasis, type 1, is associated with decreased farnesoid X receptor activity Gastroenterology 2004 126 3 756 764 10.1053/j.gastro.2003.12.013 14988830

[B13] Hayashi H Sugiyama Y 4-phenylbutyrate enhances the cell surface expression and the transport capacity of wild-type and mutated bile salt export pumps Hepatology 2007 45 6 1506 1516 10.1002/hep.21630 17538928

[B14] Hayashi H Inamura K Aida K Naoi S Horikawa R Nagasaka H Takatani T Fukushima T Hattori A Yabuki T Horii I Sugiyama Y AP2 adaptor complex mediates bile salt export pump internalization and modulates its hepatocanalicular expression and transport function Hepatology 2012 55 6 1889 1900 10.1002/hep.25591 22262466

[B15] Gonzales E Grosse B Cassio D Davit-Spraul A Fabre M Jacquemin E Successful mutation-specific chaperone therapy with 4-phenylbutyrate in a child with progressive familial intrahepatic cholestasis type 2 J Hepatol 2012 57 3 695 698 10.1016/j.jhep.2012.04.017 22609309

[B16] Naoi S Hayashi H Inoue T Tanikawa K Igarashi K Nagasaka H Kage M Takikawa H Sugiyama Y Inui A Nagai T Kusuhara H Improved liver function and relieved pruritus after 4-phenylbutyrate therapy in a patient with progressive familial intrahepatic cholestasis type 2 J Pediatr 2014 164 5 1219 1227 e1213 10.1016/j.jpeds.2013.12.032 24530123

[B17] Davit-Spraul A Fabre M Branchereau S Baussan C Gonzales E Stieger B Bernard O Jacquemin E ATP8B1 and ABCB11 analysis in 62 children with normal gamma-glutamyl transferase progressive familial intrahepatic cholestasis (PFIC): phenotypic differences between PFIC1 and PFIC2 and natural history Hepatology 2010 51 5 1645 1655 10.1002/hep.23539 20232290

[B18] Pawlikowska L Strautnieks S Jankowska I Czubkowski P Emerick K Antoniou A Wanty C Fischler B Jacquemin E Wali S Blanchard S Nielsen IM Bourke B McQuaid S Lacaille F Byrne JA van Eerde AM Kolho KL Klomp L Houwen R Bacchetti P Lobritto S Hupertz V McClean P Mieli-Vergani G Shneider B Nemeth A Sokal E Freimer NB Knisely AS Differences in presentation and progression between severe FIC1 and BSEP deficiencies J Hepatol 2010 53 1 170 178 10.1016/j.jhep.2010.01.034 20447715 PMC3042805

[B19] Klomp LW Vargas JC van Mil SW Pawlikowska L Strautnieks SS van Eijk MJ Juijn JA Pabón-Peña C Smith LB DeYoung JA Byrne JA Gombert J van der Brugge G Berger R Jankowska I Pawlowska J Villa E Knisely AS Thompson RJ Freimer NB Houwen RH Bull LN Characterization of mutations in ATP8B1 associated with hereditary cholestasis Hepatology 2004 40 1 27 38 10.1002/hep.20285 15239083

[B20] Strautnieks SS Byrne JA Pawlikowska L Cebecauerová D Rayner A Dutton L Meier Y Antoniou A Stieger B Arnell H Ozçay F Al-Hussaini HF Bassas AF Verkade HJ Fischler B Németh A Kotalová R Shneider BL Cielecka-Kuszyk J McClean P Whitington PF Sokal E Jirsa M Wali SH Jankowska I Pawłowska J Mieli-Vergani G Knisely AS Bull LN Thompson RJ Severe bile salt export pump deficiency: 82 different ABCB11 mutations in 109 families Gastroenterology 2008 134 4 1203 1214 10.1053/j.gastro.2008.01.038 18395098

[B21] Whitington PF Whitington GL Partial external diversion of bile for the treatment of intractable pruritus associated with intrahepatic cholestasis Gastroenterology 1988 95 1 130 136 3371608 10.1016/0016-5085(88)90301-0

[B22] Nakamura K Igarashi K Ide K Ohkawa R Okubo S Yokota H Masuda A Oshima N Takeuchi T Nangaku M Okudaira S Arai H Ikeda H Aoki J Yatomi Y Validation of an autotaxin enzyme immunoassay in human serum samples and its application to hypoalbuminemia differentiation Clin Chim Acta 2008 388 1–2 51 58 17963703 10.1016/j.cca.2007.10.005

[B23] Hayashi H Takada T Suzuki H Akita H Sugiyama Y Two common PFIC2 mutations are associated with the impaired membrane trafficking of BSEP/ABCB11 Hepatology 2005 41 4 916 924 10.1002/hep.20627 15791618

[B24] Kremer AE Martens JJ Kulik W Ruëff F Kuiper EM van Buuren HR van Erpecum KJ Kondrackiene J Prieto J Rust C Geenes VL Williamson C Moolenaar WH Beuers U Oude Elferink RP Lysophosphatidic acid is a potential mediator of cholestatic pruritus Gastroenterology 2010 139 3 1008 1018 1018 e1001 10.1053/j.gastro.2010.05.009 20546739

[B25] Elman S Hynan LS Gabriel V Mayo MJ The 5-D itch scale: a new measure of pruritus Br J Dermatol 2010 162 3 587 593 10.1111/j.1365-2133.2009.09586.x 19995367 PMC2875190

[B26] van der Velden LM Stapelbroek JM Krieger E van den Berghe PV Berger R Verhulst PM Holthuis JC Houwen RH Klomp LW van de Graaf SF Folding defects in P-type ATP 8B1 associated with hereditary cholestasis are ameliorated by 4-phenylbutyrate Hepatology 2010 51 1 286 296 10.1002/hep.23268 19918981

[B27] Aoki J Inoue A Okudaira S Two pathways for lysophosphatidic acid production Biochim Biophys Acta 2008 1781 9 513 518 10.1016/j.bbalip.2008.06.005 18621144

[B28] Alemi F Kwon E Poole DP Lieu T Lyo V Cattaruzza F Cevikbas F Steinhoff M Nassini R Materazzi S Guerrero-Alba R Valdez-Morales E Cottrell GS Schoonjans K Geppetti P Vanner SJ Bunnett NW Corvera CU The TGR5 receptor mediates bile acid-induced itch and analgesia J Clin Invest 2013 123 4 1513 1530 10.1172/JCI64551 23524965 PMC3613908

[B29] Kim SW Hooker JM Otto N Win K Muench L Shea C Carter P King P Reid AE Volkow ND Fowler JS Whole-body pharmacokinetics of HDAC inhibitor drugs, butyric acid, valproic acid and 4-phenylbutyric acid measured with carbon-11 labeled analogs by PET Nucl Med Biol 2013 40 7 912 918 10.1016/j.nucmedbio.2013.06.007 23906667 PMC3769509

[B30] van Mil SW Klomp LW Bull LN Houwen RH FIC1 disease: a spectrum of intrahepatic cholestatic disorders Semin Liver Dis 2001 21 4 535 544 10.1055/s-2001-19034 11745041

