# Supplementary material for: Intractable itch relieved by 4-phenylbutyrate therapy in patients with progressive familial intrahepatic cholestasis type 1
Source: Orphanet J Rare Dis. 2014 Jul 15;9:89. doi: 10.1186/1750-1172-9-89 (PMC4105841; doi:10.1186/1750-1172-9-89)
Supplement: Additional file 1 — Supplemental information. [file 1750-1172-9-89-S1.docx]

**Supplemental information**

**Intractable itch relieved by 4-phenylbutyrate therapy in patients with progressive familial intrahepatic cholestasis type 1**

Yasuhiro Hasegawa^1, *^, Hisamitsu Hayashi^2, *^, Sotaro Naoi^2, *^, Hiroki Kondou^1^, Kazuhiko Bessho^1^, Koji Igarashi^3^, Kentaro Hanada^4^, Kie Nakao^1^, Takeshi Kimura^1^, Akiko Konishi^1^, Hironori Nagasaka^5^, Yoko Miyoshi^1^, Keiichi Ozono^1^, and Hiroyuki Kusuhara^2^

(Y.H., H.H., and S.N. contributed equally to this work.)

1. Department of Pediatrics, Osaka University Graduate School of Medicine, 2-2 Yamada-oka, Suita, Osaka 565-0871, Japan
2. Laboratory of Molecular Pharmacokinetics, Graduate School of Pharmaceutical Sciences, The University of Tokyo, 7-3-1 Hongo, Bunkyo-ku, Tokyo 113-0033, Japan
3. Bioscience Division, Reagent Development Department, TOSOH Corporation, 2743-1 Hayakawa, Ayase-shi, Kanagawa 252-1123, Japan
4. Department of Biochemistry and Cell Biology, National Institute of Infectious Diseases, 1-23-1, Toyama, Shinjuku-ku, Tokyo 162-8640, Japan
5. Department of Pediatrics, Takarazuka City Hospital, 4-5-1 Kohama, Takarazuka-shi, Hyogo 665-0827, Japan

# **Methods**

## **Materials**

Pharmaceutical grade 4PB was purchased from Sigma-aldrich (St. Louis, MO) for the *in vitro* studies and from Swedish Orphan Inter AB (Stockholm, Sweden) for the treatment of the PFIC1 patients. Antibodies against HA, FLAG, and MRP2 were purchased from Roche Diagnostics (Mannheim, Germany), Sigma-aldrich (St. Louis, MO), and Enzo Life Sciences (Plymouth Meeting, PA), respectively. Antibodies against BSEP, ATP8B1, Na^+^, K^+^-ATPase α1 subunit, and calnexin were obtained from Abcam (Cambridge, UK). Alexa Fluor Secondary Antibodies were purchased from Invitrogen (Carlsbad, CA). All other chemicals were of analytical grade.

## **Cell culture**

McA-RH7777 cells were obtained from the American Type Culture Collection (ATCC Number: CRL-1601). UPS-1 cells were a kind gift from Dr. Kentaro Hanada (National Institute of Infectious Diseases, Tokyo, Japan) [[1](#_ENREF_1)]. The cells were cultured in Dulbecco’s modified Eagle’s medium (DMEM; Invitrogen) (McA-RH7777) or Ham's F12 Nutrient Mixture (F12; Invitrogen) (CHO-K1 and UPS-1) supplemented with 10% fetal bovine serum (FBS) at 37 °C in 5% CO_2_ and 95% humidity.

## **Plasmids**

cDNA of human *ATP8B1* (AF038007) was subcloned into pShuttle vector (Clontech, Palo Alto, CA). Site-directed mutagenesis was performed as described previously [[2](#_ENREF_2), [3](#_ENREF_3)] to attach the FLAG-tag to the C-terminus of human ATP8B1 (ATP8B1^wild type (WT)^–FLAG) and to introduce the c.1587–89del (p.F529del), c.234C>G (p.H78Q) and c.2021T>C (p.M674T), or c.1729A>G (p.I577V) mutation into ATP8B1^WT^–FLAG (ATP8B1^F529del^–FLAG, ATP8B1^H78Q+M674T^–FLAG, or ATP8B1^I577V^–FLAG). cDNA of human *CDC50A* (NM_018247) was amplified by PCR from cDNA of HuH7 with specific primer sets including a hemagglutinin antigen (HA) tag in the forward primer (HA–CDC50A) and subcloned into pShuttle vector.

## ***In vitro* studies**

UPS-1 and McA-RH7777 cells co-transfected with pShuttle vectors containing cDNA of ATP8B1^WT^–FLAG, ATP8B1^F529del^–FLAG, ATP8B1^H78Q+M674T^–FLAG, ATP8B1^I577V^–FLAG, or empty vector (EV) and cDNA of HA–CDC50A or EV were subjected to quantitative PCR analysis (qPCR), cell surface biotinylation, immunocytochemistry, and Annexin V assays. The cells were treated with 4PB at various concentrations, as indicated in the figures, for 24 h before the *in vitro* experiments.

## **Measurement of mRNA expression levels**

UPS-1 cells were seeded in 24-well plates at a density of 1.2 × 10^5^ cells per well, co-transfected with pShuttle vector containing cDNA of ATP8B1^WT^-FLAG, ATP8B1^F529del^-FLAG, ATP8B1^H78Q+M674T^-FLAG, ATP8B1^I577V^-FLAG cDNA, or EV using XtremeGENE HP DNA (Roche Diagnostics, Mannheim, Germany), and treated with or without 4PB at the various concentrations indicated in the figures for 24 h. RNA was isolated using Isogen II (NIPPON GENE, Tokyo, Japan) according to the manufacturer’s instructions. Total RNA from the liver specimens of humans was isolated using an RNeasy Mini Kit (Qiagen, Hilden, Germany).

Reverse transcription was performed using ReverTra Ace® qPCR RT Master Mix with gDNA Remover (TOYOBO, Osaka, Japan). ATP8B1, BSEP, and GAPDH mRNA levels were determined by quantitative PCR (qPCR) using a LightCycler and the appropriate software (Ver. 3.53; Roche Diagnostics) as described previously [[2](#_ENREF_2), [4](#_ENREF_4)]. qPCR was performed using the following primers: 5′-ATGCAAGGATGGAAAACCAG-3′ and 5′-CGCATCCGTCTTTCTTCTTC-3′ (ATP8B1), 5′-TGCCCAGTGCATCATGTTTA-3′ and 5′-CCCTGGAAGTTGTCCCATTT-3′ (BSEP), 5′-GGCCAACATACATGCCTTCATCGAG-3′ and 5′-TGTCCAGGGCTTCTTGGACAACC-3′ (P-gp), and 5′-GGGGAGCCAAAAGGGTCATCATCT-3′ and 5′-GACGCCTGCTTCACCACCTTCTTG-3′ (GAPDH). These primers amplified cDNA sequences of ATP8B1 and BSEP. Gene expression in each reaction was normalized by the expression of GAPDH in UPS-1 cells or P-gp in human liver specimens as appropriate.

## **Cell surface biotinylation**

UPS-1 cells were seeded at a density of 1.2 × 10^5^ cells per well in 6-well plates, co-transfected with pShuttle vector containing cDNA of HA-CDC50A and of ATP8B1^WT^-FLAG, ATP8B1^F529del^-FLAG, ATP8B1^H78Q+M674T^-FLAG, ATP8B1^I577V^-FLAG cDNA, or EV using XtremeGENE HP DNA, and treated with or without 4PB at the various concentration indicated in figures for 24 h. Forty-eight hours after the transfection, cell surface biotinylation was performed to investigate the expression on the plasma membrane as described previously [[2](#_ENREF_2), [4](#_ENREF_4)]. The isolated biotinylated proteins were subjected to immunoblotting.

## **Immunocytochemistry**

UPS-1 cells and McA-RH7777 cells were co-transfected with pShuttle vector containing cDNA of HA-CDC50A or EV and cDNA of ATP8B1^WT^-FLAG, ATP8B1^F529del^-FLAG, ATP8B1^H78Q+M674T^-FLAG, or ATP8B1^I577V^-FLAG using XtremeGENE HP DNA, and seeded on glass coverslips (Matsunami Glass Ind Ltd, Osaka, Japan) in 12-well plates. The cells were fixed in 4% paraformaldehyde/PBS for 10 min, permeabilized in 0.1% Saponin/PBS for 10 min, blocked with 3% BSA/PBS for 30 min, and stained with anti-FLAG, anti-HA, and anti-calnexin (ER marker) or anti-Na^+^, K^+^-ATPase α1-subunit (UPS-1 cells)/anti-MRP2 (McA-RH7777 cells) (plasma membrane marker) for 2 h followed by Alexa Fluor 488 donkey anti-goat immunoglobulin G, Alexa Fluor 647 donkey anti-rat immunoglobulin G, and Alexa Fluor 546 donkey anti-rabbit (ER marker) or anti-mouse (plasma membrane/canalicular membrane marker) immunoglobulin G for 1 h. These staining procedures were performed at room temperature. After being mounted onto glass slides with VECTASHIELD mounting medium (Vector Laboratories Inc., Burlingame, CA), cells were visualized by confocal microscopy using a Leica TCS SP5 II laser scanning confocal microscope (Leica, Solms, Germany).

## **Annexin V assay**

Annexin V assays were conducted as reported previously [[5](#_ENREF_5)] with minor modifications. UPS-1 cells were plated into 6-cm dishes and co-transfected with pShuttle vector containing cDNA of HA-CDC50A and of ATP8B1^WT^-FLAG, ATP8B1^F529del^-FLAG, ATP8B1^H78Q+M674T^-FLAG, ATP8B1^I577V^-FLAG, or EV using XtremeGENE HP DNA. Forty-eight hours after the transfection, the transfected cells were trypsinized, washed, and incubated for 2 h at 37 °C in 1 ml of F12 with 10% FBS. Then, the cells were washed with incubation medium (132 mM NaCl, 6 mM KCl, 1 mM MgSO_4_, 1.2 mM potassium phosphate buffer, pH 7.4 (Kpi), 20 mM 4-(2-hydroxyethyl)-1-piperazine ethanesulfonic acid (pH 7.4), 10mM glucose, and 0.5% human serum albumin), resuspended in an incubation medium supplemented with 2.5 mM CaCl_2_, and incubated on ice for 0.5 h with 50 μg/ml propidum iodide (PI) with or without FITC-labeled Annexin V (FITC-Annexin V). The cells were analyzed on a BD FACSAria II Cell Sorter (BD Biosciences, San Jose, CA). Dead cells stained with PI were excluded from the analysis. FITC-Annexin V positive cells were defined as the cells that were stained with FITC-Annexin V and then showed a higher FITC signal than the cells not stained with FITC-Annexin V.

## **Statistical analysis**

The data in the figures are presented as the mean ± standard error (SE). The significance of differences between two variables and multiple variables was calculated at the 95% confidence level using Student’s t test and one-way ANOVA with Tukey’s test, respectively, using Prism software (GraphPad Software, Inc., La Jolla, CA).

# **Supplemental results**

## **Characterization of the mutations in *ATP8B1***

Characterization of the mutations in *ATP8B1* was carried out in UPS-1 cells, a Chinese hamster ovary mutant cell line that was previously used to determine the expression, cellular localization, and PS flippase activity of ATP8B1 [[1](#_ENREF_1), [6](#_ENREF_6)], and in McA-RH7777 cells, a rat hepatoma cell line that forms bile canaliculi-like structures through the formation of hepatocyte-like couplets [[2](#_ENREF_2), [7](#_ENREF_7)]. As reported previously, when co-expressed with HA–CDC50A, which stabilizes ATP8B1 and facilitates its correct trafficking to the plasma membrane through formation of a complex with ATP8B1 [[6](#_ENREF_6)], ATP8B1^WT^–FLAG was observed on the plasma membrane of both cell lines (Supplemental Fig. 1B, C) and stimulated the internalization of endogenous PS in the outer leaflet of the plasma membrane (Supplemental Fig. 1D). The PS flippase activity was determined by the cell surface binding of fluorescein isothiocyanate (FITC)-labeled Annexin V (FITC–Annexin V), which recognizes and binds to PS in outer leaflet of the plasma membrane. Ectopic expression of ATP8B1^WT^–FLAG significantly decreased the number of FITC–Annexin V-positive cells (Supplemental Fig. 1D). However, introduction of p.F529del into ATP8B1 significantly decreased cell surface expression of ATP8B1 without affecting its mRNA expression (Supplemental Fig. 1A, B), resulting in no reduction in FITC–Annexin V-positive cells (Supplemental Fig. 1D). The decreased cell surface expression of ATP8B1^F529del^ was confirmed by immunocytochemistry that showed no colocalization with the plasma membrane marker, NaKα1, in UPS-1 cells and with the canalicular membrane marker, MRP2, in McA-RH7777 cells (Supplemental Fig. 1C). By contrast, introduction of p.H78Q and p.M674T mutations or p.I577V mutation into ATP8B1^WT^–FLAG cDNA had no effect on mRNA and protein expression, trafficking to the plasma membrane, or PS flippase activity of ATP8B1^WT^–FLAG (Supplemental Fig. 1A–D). Therefore, it is likely that the decreased mRNA and protein expression of ATP8B1 in patient 3 is caused by mutations in the promoter region and/or UTR of *ATP8B1* that affect transcription of *ATP8B1* and stabilization of ATP8B1 mRNA, but not by the mutations analyzed in this study.

# **Supplemental figure legends**

## **Supplemental Fig. S1. Effects of mutations in *ATP8B1* on mRNA and protein expression levels, cellular localization, and function of ATP8B1**

UPS-1 cells (A–D) and McA-RH7777 cells (C) were transfected with pShuttle vector containing cDNA of ATP8B1^WT^–FLAG, ATP8B1^F529del^–FLAG, ATP8B1^H78Q+M674T^–FLAG, ATP8B1^I577V^–FLAG, or EV together with (B–D) or without (A, C) pShuttle vector containing HA–CDC50A cDNA. (A, B) Determination of mRNA and protein expression. The cells were subjected to qPCR (A) and cell surface biotinylation (B), and then analyzed as described in the Supplementary Material. In (B), the band intensities of FLAG (140 kDa; mature form of ATP8B1-FLAG) were quantified. The signal intensity is presented below each panel. (C) Cellular localization. The cells were subjected to immunocytochemistry and analyzed by confocal immunofluorescence microscopy as described in the Supplementary information. White in the merged images indicates colocalization of FLAG, HA, and MRP2. Scale bar: 10 μm. (D) Determination of PS flippase activity. The cells were subjected to an Annexin V assay. The percentage of PS positive cells was calculated as described in the Supplementary information. *, *p*<0.05, **, *p*< 0.01. In (A–D), a representative result of three independent experiments is shown. Bars represent the mean ± SE of each experiment in triplicate. AU, arbitrary unit; GAPDH, glyceraldehyde-3-phosphate dehydrogenase; ND, not detected because of low expression.

## **Supplemental Fig. S2. Effect of 4PB on the expression levels of ATP8B1 mutants**

UPS-1 cells were transfected with pShuttle vector containing cDNA of ATP8B1^WT^–FLAG, ATP8B1^F529del^–FLAG, ATP8B1^H78Q+M674T^–FLAG, or ATP8B1^I577V^–FLAG together with pShuttle vector containing HA–CDC50A cDNA, treated with 4PB at the indicated concentration for 24 h, and then subjected to cell surface biotinylation as described in the Supplemental information. The biotinylated cells were lysed (input), precipitated with streptavidin–agarose beads, eluted from the beads (elute), and analyzed by immunoblotting. The FLAG signal intensity of the experiment depicted was corrected for protein loading using NaKα1 expression and is presented relative to the average of the control condition below each panel. A representative image of three independent experiments is shown. AU, arbitrary unit; ND, not detected.

# **References**

[1] Hanada K, Pagano RE. A Chinese hamster ovary cell mutant defective in the non-endocytic uptake of fluorescent analogs of phosphatidylserine: isolation using a cytosol acidification protocol. The Journal of cell biology 1995;128:793-804.

[2] Hayashi H, Inamura K, Aida K, Naoi S, Horikawa R, Nagasaka H, et al. AP2 adaptor complex mediates bile salt export pump internalization and modulates its hepatocanalicular expression and transport function. Hepatology (Baltimore, Md 2012;55:1889-1900.

[3] Hayashi H, Takada T, Suzuki H, Akita H, Sugiyama Y. Two common PFIC2 mutations are associated with the impaired membrane trafficking of BSEP/ABCB11. Hepatology (Baltimore, Md 2005;41:916-924.

[4] Hayashi H, Mizuno T, Horikawa R, Nagasaka H, Yabuki T, Takikawa H, et al. 4-Phenylbutyrate modulates ubiquitination of hepatocanalicular MRP2 and reduces serum total bilirubin concentration. Journal of hepatology 2012;56:1136-1144.

[5] Klomp LW, Vargas JC, van Mil SW, Pawlikowska L, Strautnieks SS, van Eijk MJ, et al. Characterization of mutations in ATP8B1 associated with hereditary cholestasis. Hepatology (Baltimore, Md 2004;40:27-38.

[6] Paulusma CC, Folmer DE, Ho-Mok KS, de Waart DR, Hilarius PM, Verhoeven AJ, et al. ATP8B1 requires an accessory protein for endoplasmic reticulum exit and plasma membrane lipid flippase activity. Hepatology (Baltimore, Md 2008;47:268-278.

[7] Aida K, Hayashi H, Inamura K, Mizuno T, Sugiyama Y. Differential roles of ubiquitination in the degradation mechanism of cell surface-resident bile salt export pump and multidrug resistance-associated protein 2. Molecular pharmacology 2014;85:482-491.

# **Supplemental Table S1**

List of drugs given to the patients before, during, and after the course of this study
