# Supplementary figures and images for: Intractable itch relieved by 4-phenylbutyrate therapy in patients with progressive familial intrahepatic cholestasis type 1
Source: Orphanet J Rare Dis. 2014 Jul 15;9:89. doi: 10.1186/1750-1172-9-89 (PMC4105841; doi:10.1186/1750-1172-9-89)

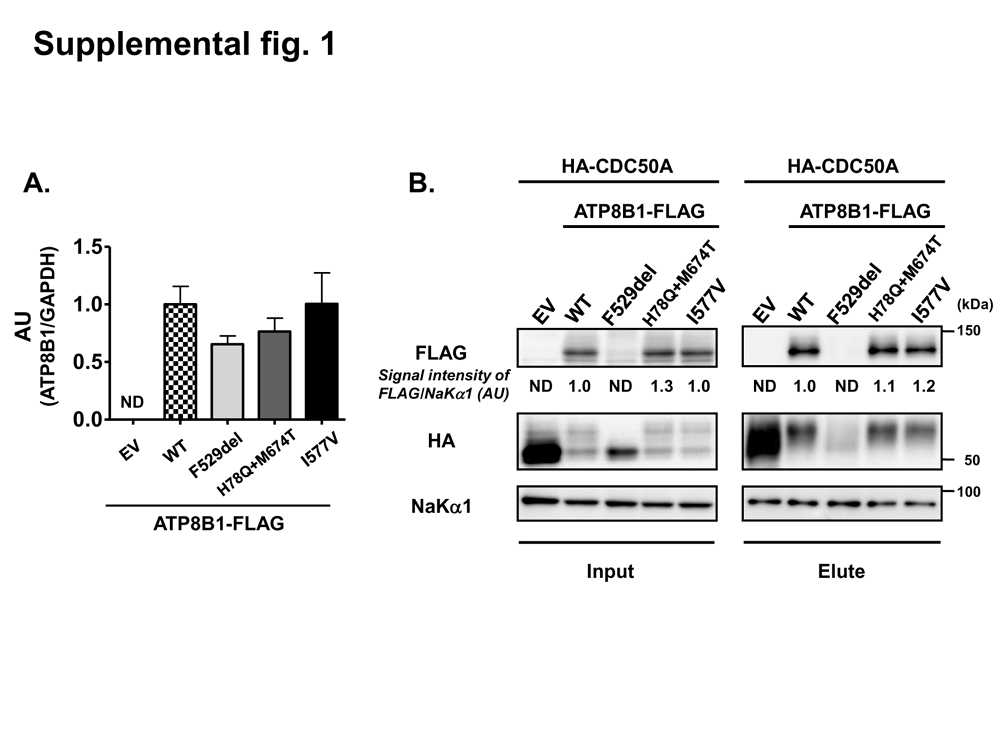

Supplement: Additional file 2 — Effects of mutations in ATP8B1 on mRNA and protein expression levels, cellular localization, and function of ATP8B1. [file 1750-1172-9-89-S2.zip › 7753618851254322_MOESM2_ESM/7753618851254322_add2.tiff]

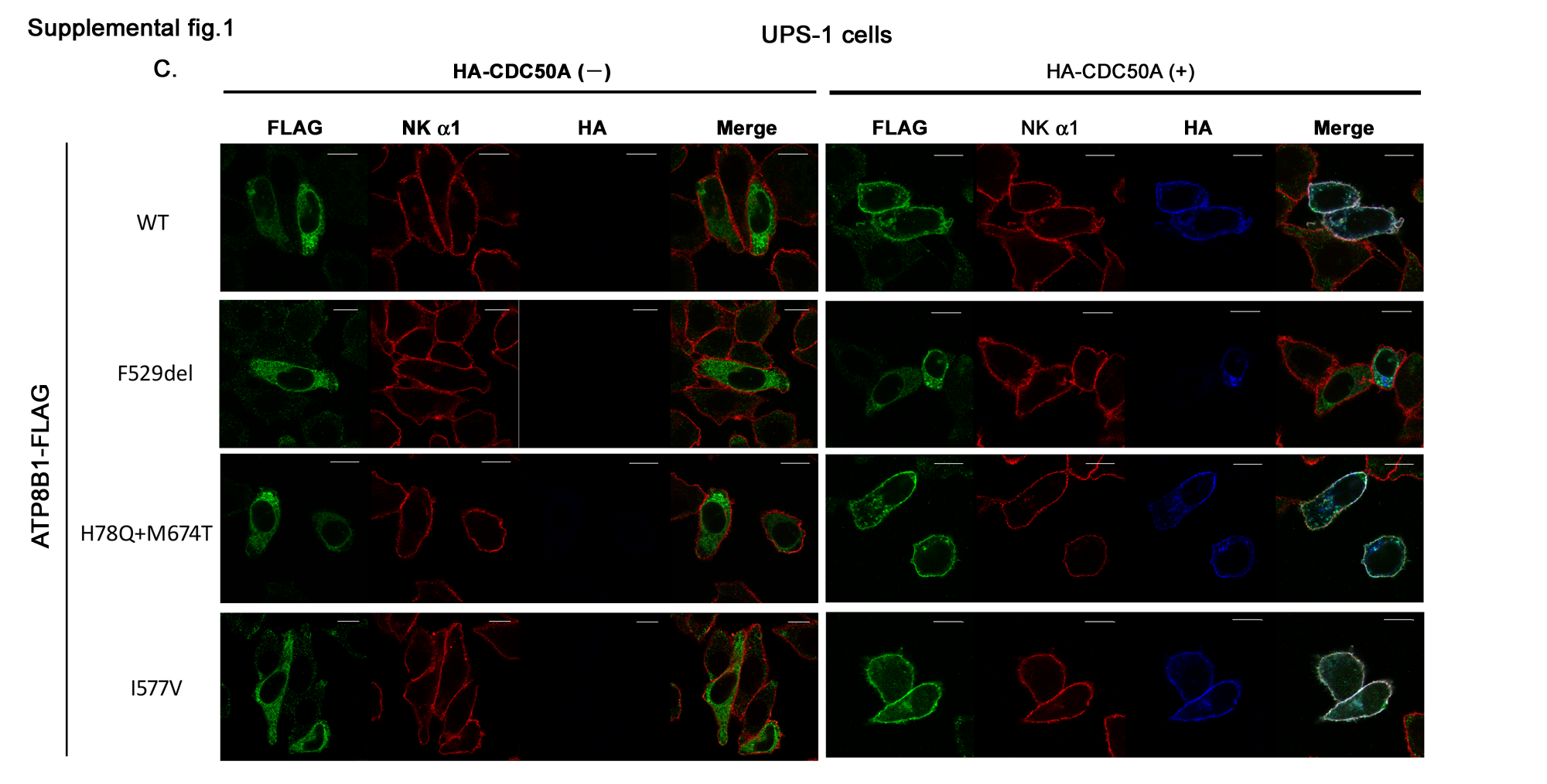

Supplement: Additional file 2 — Effects of mutations in ATP8B1 on mRNA and protein expression levels, cellular localization, and function of ATP8B1. [file 1750-1172-9-89-S2.zip › 7753618851254322_MOESM2_ESM/7753618851254322_add3.tiff]

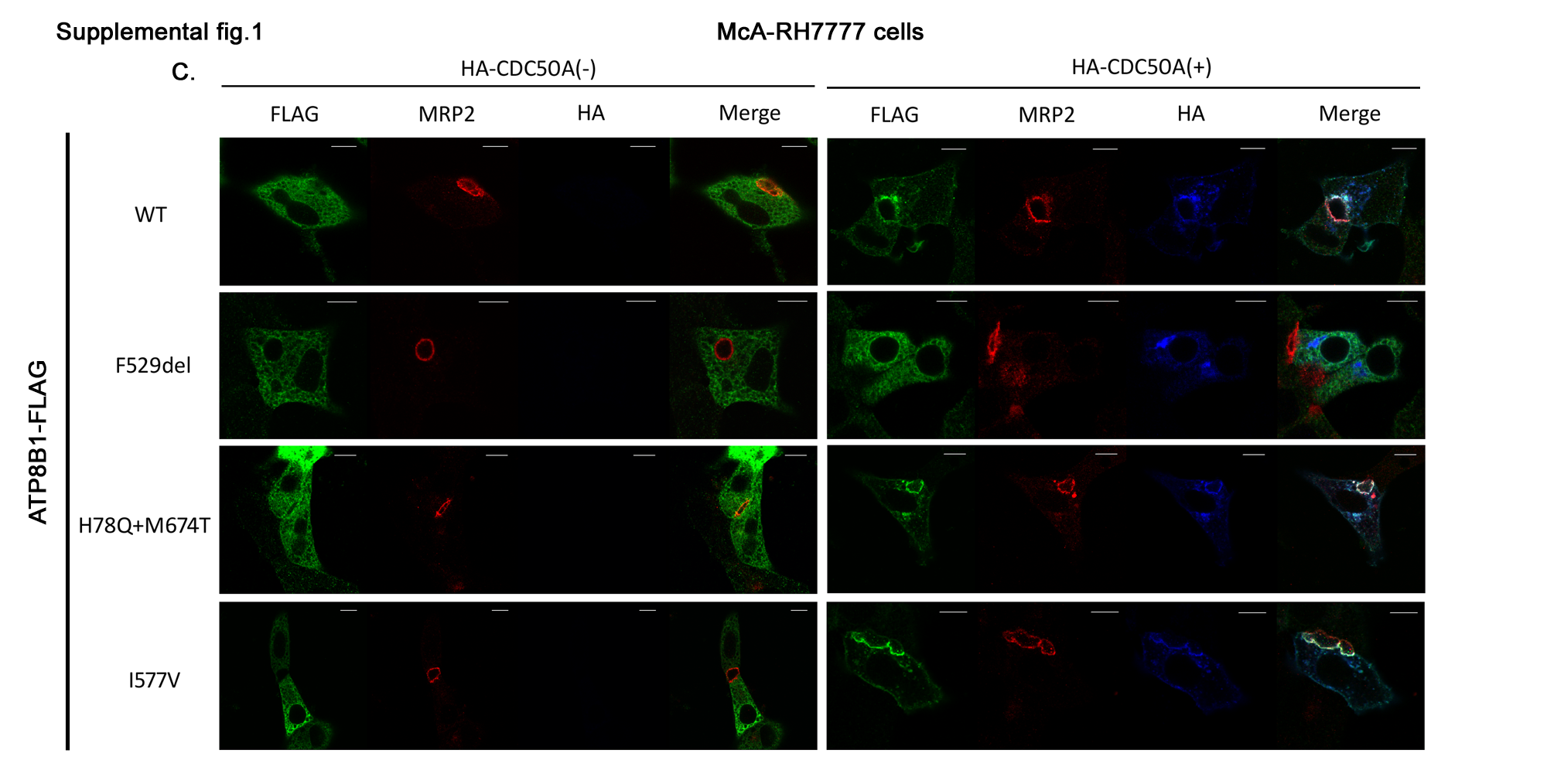

Supplement: Additional file 2 — Effects of mutations in ATP8B1 on mRNA and protein expression levels, cellular localization, and function of ATP8B1. [file 1750-1172-9-89-S2.zip › 7753618851254322_MOESM2_ESM/7753618851254322_add4.tiff]

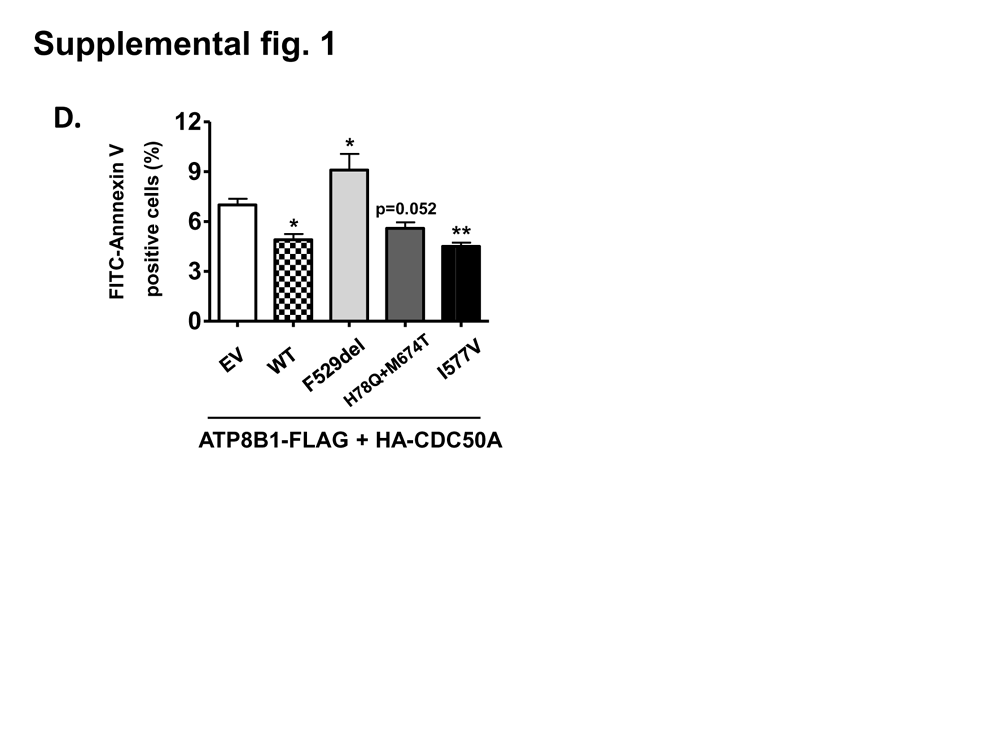

Supplement: Additional file 2 — Effects of mutations in ATP8B1 on mRNA and protein expression levels, cellular localization, and function of ATP8B1. [file 1750-1172-9-89-S2.zip › 7753618851254322_MOESM2_ESM/7753618851254322_add5.tiff]

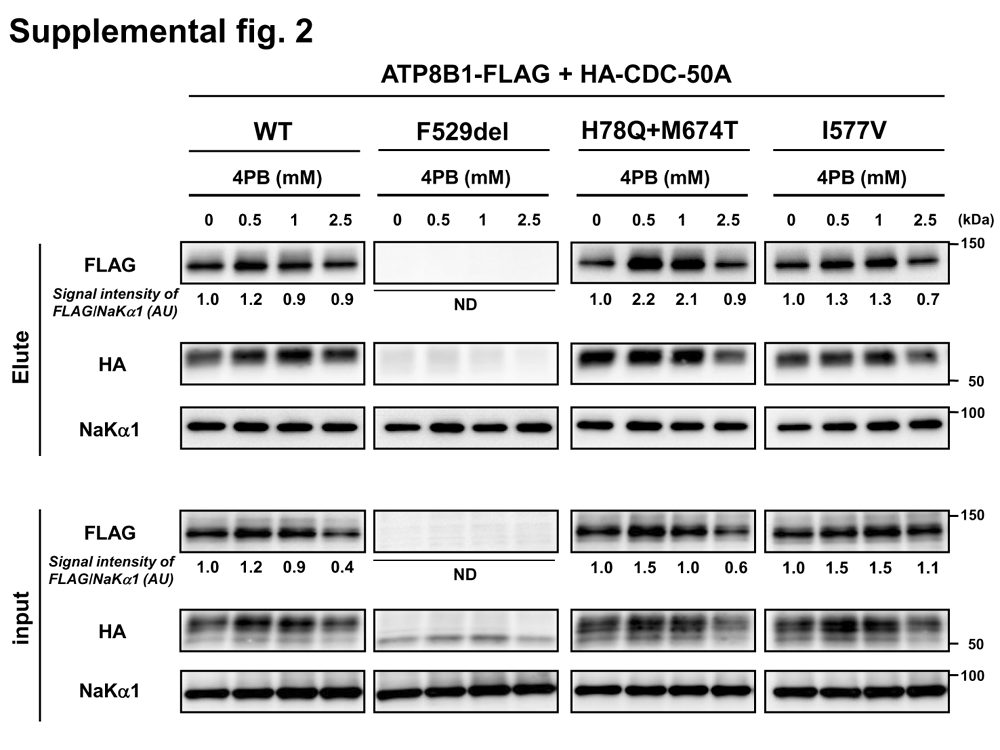

Supplement: Additional file 3 — Effect of 4PB on the expression levels of ATP8B1 mutants. [file 1750-1172-9-89-S3.tiff]
